# Evaluating the X Chromosome-Specific Diversity of Colombian Populations Using Insertion/Deletion Polymorphisms

**DOI:** 10.1371/journal.pone.0087202

**Published:** 2014-01-31

**Authors:** Adriana Ibarra, Tomás Restrepo, Winston Rojas, Adriana Castillo, António Amorim, Beatriz Martínez, German Burgos, Henry Ostos, Karen Álvarez, Mauricio Camacho, Zuleyma Suarez, Rui Pereira, Leonor Gusmão

**Affiliations:** 1 IdentiGEN - Genetic Identification Laboratory and Research Group of Genetic Identification, Institute of Biology, School of Natural and Exact Sciences (FCEN), University of Antioquia, Medellin, Antioquia, Colombia; 2 Laboratory of Molecular Genetics, Institute of Biology, University of Antioquia, Medellin, Antioquia, Colombia; 3 Laboratorio de Genética, Universidad Industrial de Santander (UIS), Bucaramanga, Santander, Colombia; 4 IPATIMUP - Institute of Molecular Pathology and Immunology of the University of Porto, Porto, Portugal; 5 FCUP - Faculty of Sciences of the University of Porto, Porto, Portugal; 6 Molecular Genetics Laboratory, Institute for Immunological Research, University of Cartagena, Cartagena, Bolivar, Colombia; 7 Molecular Genetics Laboratory, Cruz Vital, Ecuadorian Red Cross, Quito, Ecuador; 8 Genomic Medicine Laboratory, Health Faculty, Surcolombiana University, Neiva, Huila, Colombia; 9 Institute of Legal Medicine and Forensic Sciences, Northeast Regional, Arauca, Colombia; 10 Clinical Laboratory Olga Zuleima Suárez Molina, Cucuta, Norte de Santander, Colombia; 11 DNA Diagnostic Laboratory (LDD), State University of Rio de Janeiro (UERJ), Rio de Janeiro, Brazil; Universitat Pompeu Fabra, Spain

## Abstract

The European and African contribution to the pre-existing Native American background has influenced the complex genetic pool of Colombia. Because colonisation was not homogeneous in this country, current populations are, therefore, expected to have different proportions of Native American, European and African ancestral contributions. The aim of this work was to examine 11 urban admixed populations and a Native American group, called Pastos, for 32 X chromosome indel markers to expand the current knowledge concerning the genetic background of Colombia. The results revealed a highly diverse genetic background comprising all admixed populations, harbouring important X chromosome contributions from all continental source populations. In addition, Colombia is genetically sub-structured, with different proportions of European and African influxes depending on the regions. The samples from the North Pacific and Caribbean coasts have a high African ancestry, showing the highest levels of diversity. The sample from the South Andean region showed the lowest diversity and significantly higher proportion of Native American ancestry than the other samples from the North Pacific and Caribbean coasts, Central-West and Central-East Andean regions, and the Orinoquian region. The results of admixture analysis using X-chromosomal markers suggest that the high proportion of African ancestry in the North Pacific coast was primarily male driven. These men have joined to females with higher Native American and European ancestry (likely resulting from a classic colonial asymmetric mating type: European male x Amerindian female). This high proportion of male-mediated African contributions is atypical of colonial settings, suggesting that the admixture occurred during a period when African people were no longer enslaved. In the remaining regions, the African contribution was primarily female-mediated, whereas the European counterpart was primarily male driven and the Native American ancestry contribution was not gender biased.

## Introduction

The study of the genetic diversity of human populations is important to reveal different aspects associated with the history of these individuals, which, in turn, is highly significant in many applied fields, namely clinical and forensic genetics. In the clinical field, genetic research studies are designed to identify associations between some alleles and/or genotypes and diseases in different areas [1]–[3]. In this context, it is important to know the genetic composition of the concerned populations, as the results of association studies are sensitive to population substructures that can induce spurious associations between alleles at different loci [4], [5]. In the forensic field, the interpretation of genetic evidence depends on probabilities calculated on the basis of the genetic composition of the reference population [6]–[8]. Therefore, in human genetics, it is important to have a comprehensive knowledge of the genetic profiles of populations for the correct interpretation of the data.

America was the last continent to be colonised by people from Northeast Asia. After arriving in North America, there were at least three subsequent dispersions responsible for the colonisation of the continent, from North to South, reaching the most southern regions in South America [9]–[11]. The long-lasting process of colonisation throughout the most remote regions of the continent was responsible for the emergence of a large number of ethnic groups that became well differentiated in terms of their language, culture and genetic background [12]. The history of the Native American groups in the North, Central and South American subcontinents has been the focus of many genetic studies [10], [11], [13], [14]. Nevertheless, these studies have faced important challenges because much of the ancient genetic diversity has been eradicated from the extant populations. In addition, during the last five centuries, most American populations have been exposed to admixture events involving pre-existing Native groups and European and African people.

In an attempt to identify important signs of American colonisation and characterise the history of present-day populations, different types of genetic markers were used, including autosomal, mtDNA, and Y- and X chromosome-specific polymorphisms (e.g. [13]–[19]).

Similar to most populations in South America, Colombia has a complex history of colonisation, harbouring a gene pool comprising not only the contributions of different Native American groups but also great contributions from European and African people.

The genetic study of the populations in Colombia has been the focus of previous studies, using different types of markers in different populations or regions of the country [20]–[24]. Concerning the maternal lineages, the available studies have shown that most Native and admixed Colombian populations harbour a gene pool that is composed essentially by Native American haplogroups [23], [25], [26]. The African haplogroup L is the second most represented, reaching higher frequencies than the Native American haplogroups in some African descent populations in Colombia [23], [27]. In contrast, the Y chromosome specific lineages are mostly from Europe; Native American haplogroups are not common in the admixed populations but they are frequent in Native groups [23]. European haplogroups are also the most frequent ones in Colombian African descent populations, together with lineages from African origin [28], [29]. A high heterogeneity exists when analysing results based on autosomal markers, with different proportions of African, European and Native American ancestries’ depending on the region of the country [23]. So far, these studies have shown that Colombia does not present a uniform genetic pool [23], [26], [29], although many populations still need to be investigated. When evaluating the population substructure or inferring ancestry in different population subgroups, various factors, including sampling strategies and the number and/or type of markers used, can produce differences in the results. When properly integrated, the results from different population subgroups and markers will contribute to a comprehensive view of the genetic history of populations.

Indels are length variation polymorphisms produced by the insertion or deletion of one or more nucleotides. Indels are abundant, representing approximately 20% of all polymorphisms in the human genome [30]–[32]. These polymorphisms present similar mutation rates as SNPs and a low recurrence, which makes these markers more suitable to investigate population admixture events. The simplicity of indel genotyping is also an advantage in the study of large population sample sets.

The use of autosomal markers is typically the best strategy to disclose admixture patterns in populations, as these markers account for both paternal and maternal inheritance, unlike the results from linked uniparental markers. Although markers on the X chromosome represent the differential female versus male contribution, these markers can nevertheless be useful to reveal differences in populations that present higher maternal differentiation.

The aim of the present study was to examine a set of 32 X-indel markers to improve the current knowledge concerning the genetic background of Colombian populations. The typing of these markers in different populations from Colombia revealed differences among the admixture processes in the studied populations, based on genetic distances, ancestry proportions and linkage disequilibrium patterns.

## Materials and Methods

### Ethics Statement

Written informed consent was obtained from all participants for cooperation in this study under strictly confidential conditions. The present study was approved through the Ethics Committee at the University of Antioquia (Comité de Bioética, Sede Investigación Universitaria, CBEIH-SIU; Acta de aprobación: 07-42-100), in accordance with the ethical principles of the 2000 Helsinki Declaration of the World Medical Association (http://www.uma.net/e/policy/b3.htm).

### Sample Collection and DNA Extraction

**Figure 1:**
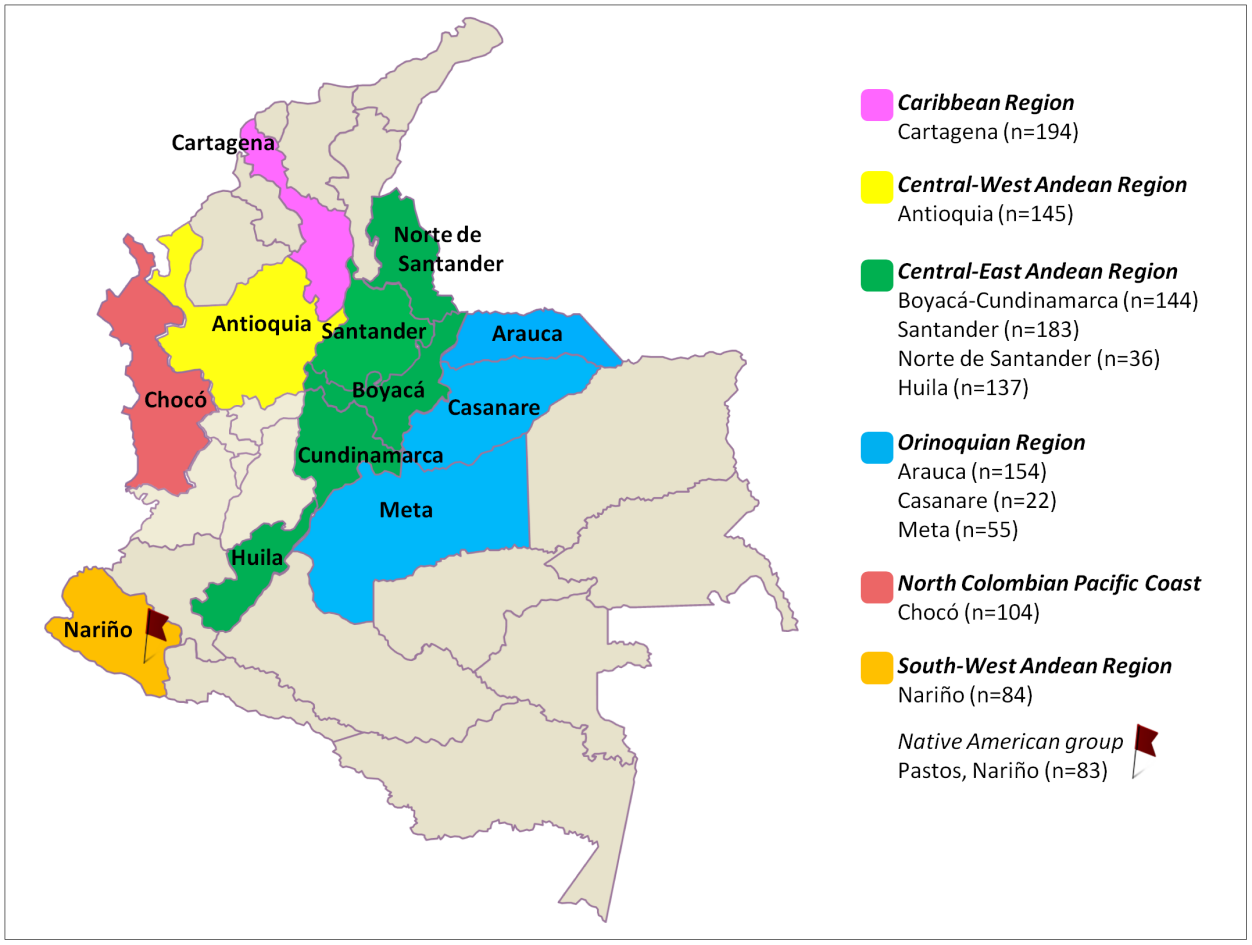
Map showing the departments of Colombia and the location of the samples included in the present study. The sample sizes (n = total number of chromosomes) are indicated for each population.

In this study, a total of 869 samples were collected using blood and buccal swabs obtained from the unrelated individuals (397 males and 472 females) in 12 different Colombian population groups (see Figure 1 for the locations and number of chromosomes per population). The DNA samples from unrelated individuals born and living in the departments of Boyacá-Cundinamarca, Antioquia, Chocó and Santander were selected from routine paternity cases. Samples from Norte de Santander, Huila, Arauca, Nariño and the Native American group of Pastos were collected from unrelated individuals with four grandparents born in one of the concerned districts. The samples from Cartagena were random samples collected as controls in a previous association study [33]. The GenMol group provided the samples from Casanare and Meta, which were used in a previous study [23].

The blood samples were collected in tubes containing EDTA and placed on Whatman™ FTA™ Gene cards (GE Healthcare Life Sciences, Buckinghamshire, UK).

Genomic DNA was extracted using a standard salting-out procedure [34] or resin Chelex 100 [35]. The DNA concentrations were determined using a Lambda Bio 10 spectrophotometer (Perkin Elmer, Waltham, MA, USA).

### Genetic Markers and Genotyping

All samples were typed for a panel of 32 X-indels using a previously described protocol [36]. The amplification of the 32 X-Indels was performed in a single PCR multiplex reaction, using 1X Qiagen multiplex PCR master mix (Qiagen), 1X primer mix and 0.3–5 ng of genomic DNA in a 10 µL final reaction volume. The sequences and final concentration of the primers in the PCR are detailed in Pereira et al. [36]. Thermal cycling conditions consisted of an initial step at 95°C for 15 min; 30 cycles at 94°C for 30 sec, 60°C for 90 sec, and 72°C for 45 sec; and a final extension at 72°C for 60 min.

The PCR products were prepared for subsequent analysis, adding 1 µL of amplified product to 12 µL of Hi-Di™ Formamide (Applied Biosystems (AB), Foster City, CA) and 0.15 µL of internal size standard GeneScan™ 500 LIZ® (AB). Capillary electrophoresis and detection were performed on a 3130 Series Genetic Analyser using a G5 filter set and POP-4™ (AB) polymer. The resulting electropherograms were analysed, and the genotypes were assigned using GeneMapper v3.2 (AB).

### Statistical Analyses

The gene diversity values, observed and expected heterozygosities, Linkage Disequilibrium and Hardy-Weinberg equilibrium tests were all calculated using ARLEQUIN software v3.5.1.3 [37]. Population comparisons by mean of the pairwise genetic distances (*F*_ST_) and the Analysis of Molecular Variance (AMOVA), as well as the corresponding non-differentiation *p*-values, were assessed using ARLEQUIN software v3.5.1.3 [37]. For an easier visualization of the observed genetic distances, a multidimensional scaling (MDS) plot of the pairwise *F*_ST_ matrix was represented using STATISTICA v7.0 software (Statsoft, Tulsa, Oklahoma; http://www.statsoft.com/).

For comparison purposes and inferences on admixture, genotypic data from African and European reference samples were obtained from a previous study including the same 32 X-indels [36].

The apportionment of genetic ancestral contributions was estimated using the STRUCTURE v2.3.3 software [38], [39]. To estimate the ancestral membership proportions in the studied populations, a supervised analysis was performed using prior information on the geographic origin of the reference samples, accounting for linkage (two blocks MID356-MID357 and MID3690-MID3719-MID2089 were considered following previous results from Pereira et al. [36]). Considering the historical formation of Colombia, we assumed an essentially tri-hybrid contribution from Native Americans, Europeans and Africans (i.e., K = 3). The STRUCTURE runs comprised three replicates of 100,000 burnin steps followed by 100,000 Markov Chain Monte Carlo (MCMC) iterations. The “*Use population Information to test for migrants*” option was used with the Linkage model; the allele frequencies were correlated and updated using only individuals with POPFLAG = 1, in this case the African, European [36] and Pastos Native American samples were used as ancestral reference populations.

## Results and Discussion

The 32 X-indel profiles obtained for the 869 Colombian samples are listed in Table S1.

### Population Pairwise Differences and AMOVA

The allele frequencies were calculated in both male and female samples, and no significant differences were observed. Thus, all chromosomes from males and females were pooled in a single sample for each population and used for population comparisons.

**Figure 2:**
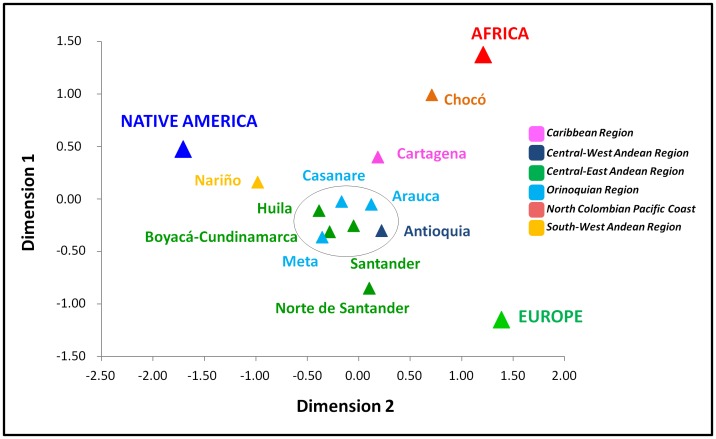
Multidimensional scaling plot of the pairwise *F*_ST_ genetic distances calculated between samples from Africa, Europe, and different Colombian populations, including a sample from Native Americans (Pastos) (Stress = 0.0556). The circle in the centre of the plot is grouping the samples with no significant population differentiation *p-values* detected (see Table S2).

A comparison was made between all samples from the 12 populations in Colombia and two published reference samples from Europe and Africa [36]. Pairwise genetic distances and respective non-differentiation *p*-values are listed in Table S2. To improve the visualisation of the results, the pairwise genetic distances were represented in a two-dimensional MDS plot (Figure 2). The addition of a third dimension to the MDS plot did not reveal important differences (data not shown), and the stress value (0.0379) was just slightly lower than that obtained for the two-dimensional representation (0.0556). The adequacies of the two-dimensional MDS plot to represent the input genetic distances’ data is also reinforced by the Shepard Diagram (Figure S1).

Significant differences were detected between the samples from the Native American group of Pastos and all admixed populations, except Nariño and Casanare. The genetic distance between Pastos and Casanare is nevertheless high, indicating that the observed result can be due to the small sample size of Casanare.

Significant differences were also observed in all pairwise comparisons of the 12 different samples from the Colombian populations and the reference samples from Europe and Africa.

Comparisons between pairs of Colombian admixed population samples showed significant differentiation values between the Chocó, Cartagena and Nariño populations in most pairwise comparisons with the remaining samples. In eleven pairwise comparisons, significant differences were found between Chocó and nine other Colombian samples. Cartagena and Nariño showed a significant differentiation in six out of eleven comparisons (see Table S2). Chocó is expected to harbour a significant African ancestry [23], [26], consistent with the observed lower genetic distance to the African sample used as a reference. Although more distant to the reference African sample, Cartagena also showed a higher African affinity than the other population samples. The admixed population sample from Nariño presents a lower genetic distance to the Native American group from the same region than any other sample.

Moreover, the position of Norte de Santander in the plot was displaced in a direction that did not correspond to any of the three African, Native American and European source populations. This sample was expected to group with the samples from the Central-East Andean region, which was not the case. Nevertheless, the observed values for the genetic distance were not high enough to exclude the hypothesis that no significant differences exist between Norte de Santander and the other three populations from the Central-East Andean region (Table S2). In summary, this result most likely reflects the small size of the sample from Norte de Santander. Thus, the Norte the Santander sample was excluded when calculating the allele frequencies in the Central-East Andean region.

Because differences were not observed between the samples from Boyacá-Cundinamarca, Huila and Santander, all located in the Central-East Andean region and the samples from Arauca, Meta and Casanare in the Orinoquian region, these samples were grouped. Subsequently, all samples were classified according to the geographic region using the same criteria as in previous studies [20], [40].

After grouping the samples according to geographic region, AMOVA revealed a significant proportion of variation between groups, with a fixation index *F*_CT_ of 0.0142 (non-differentiation *p*-value = 0.0065±0.0008). A low percentage of variation was observed among populations within groups (*F*_SC_ = 0.0021; non-differentiation *p*-value = 0.0885±0.0027). This result demonstrates the consistency of the groups considered.

### Genetic Characterisation of the Diversity in the Populations Studied

The generated data were used to test Hardy-Weinberg equilibrium in the female samples. For a significance level of 0.00156, obtained using Bonferroni’s correction for multiple tests (32 per population), no significant deviations for the genotypic distributions were observed within any population (the *p-*values were all above 0.0044 for a total of 384 tests, for 32 markers tested in 12 populations). The Hardy-Weinberg equilibrium was also tested after pooling the female samples from the Central-East Andean and Orinoquian regions, and no statistically significant deviations were detected from the expected genotypic distributions in any of the tested regions in Colombia (data not shown).

The allele frequencies and genetic diversity values for the 32 loci were calculated in the samples from each Colombian region (Table 1 and Table S3). The highest diversity values were observed for the samples from the Caribbean and North Pacific coast. The lowest diversity values were observed for the Native group of Pastos. The samples from the Central-West and Central-East Andean regions and from the Orinoquian region presented similar diversity values, which were slightly higher than the values obtained in the South-West Andean region.

**Table 1:** Average gene diversity values among the 32 X-indel loci in the samples obtained from the Native American group (Pastos) and the six Colombian regions: South-West Andean Region (Nariño); Central-West Andean Region (Antioquia); Central-East Andean Region (Boyacá-Cundinamarca, Huila and Santander); Orinoquian Region (Arauca, Meta and Casanare); North Colombian Pacific Coast (Chocó); and the Caribbean Region (Cartagena).

Region	n	Average gene diversity values over loci
Central-West Andean	145	0.3955±0.1984
Central-East Andean	464	0.3930±0.1964
Orinoquian	231	0.3938±0.1972
Caribbean	194	0.4237±0.2115
North Colombian Pacific Coast	104	0.4148±0.2081
South-West Andean	84	0.3618±0.1833
Native American group	83	0.3196±0.1632

### Linkage Disequilibrium

Pairwise linkage disequilibrium (LD) was tested in male samples from each Colombian region. Table 2 lists the results obtained for all loci pairs separated by no more than 1 Mb. For more distant pairs of loci, the significant *p*-values for the LD test are listed in Table S4 (the remaining non-significant results are not shown).

**Table 2:** Exact test *p-*values of linkage disequilibrium for the polymorphic loci not separated more than 1 Mb.

Loci pair	Distance (bp)	Central West Region	Central East Region	Orinoquian Region	Caribbean Region	North Pacific Coast	South Andean Region	Native Americans
MID357-MID356	5.187	**≤5.E-7** *	**≤5.E-7** *	**≤5.E-7** *	**≤5.E-7** *	**≤5.E-7** *	**≤5.E-7** *	4.1E-4**
MID3690-MID3719	56.862	6.9E-4**	**6.E-05** *	>1.E-2	1.8E-3**	5.6E-3**	>1.E-2	>1.E-2
MID3703-MID3774	97.700	1.E-2**	**≤5.E-7** *	2.3E-3**	>1.E-2	>1.E-2	6.9E-3**	>1.E-2
MID3719-MID2089	117.035	7.2E-3**	**≤5.E-7** *	**9.9E-6** *	3.8E-3**	**≤5.E-7** *	4.1E-3**	>1.E-2
MID3690-MID2089	173.897	>1.E-2	>1.E-2	>1.E-2	>1.E-2	>1.E-2	>1.E-2	>1.E-2
MID3712-MID357	340.665	>1.E-2	>1.E-2	>1.E-2	>1.E-2	>1.E-2	>1.E-2	>1.E-2
MID3712-MID356	345.852	>1.E-2	>1.E-2	>1.E-2	>1.E-2	>1.E-2	>1.E-2	>1.E-2
MID3740-MID3732	425.269	>1.E-2	>1.E-2	>1.E-2	>1.E-2	>1.E-2	>1.E-2	>1.E-2
MID3722-MID1361	592.358	>1.E-2	>1.E-2	>1.E-2	>1.E-2	>1.E-2	>1.E-2	***
MID3736-MID3753	784.625	>1.E-2	>1.E-2	>1.E-2	>1.E-2	>1.E-2	***	***
MID356-MID3703	793.252	>1.E-2	6.7E-3**	>1.E-2	>1.E-2	>1.E-2	>1.E-2	>1.E-2
MID357-MID3703	798.439	>1.E-2	6.E-3**	>1.E-2	>1.E-2	>1.E-2	>1.E-2	>1.E-2
MID3732-MID3727	833.674	>1.E-2	>1.E-2	>1.E-2	>1.E-2	>1.E-2	>1.E-2	>1.E-2
MID356-MID3774	890.952	>1.E-2	5.8E-4**	>1.E-2	>1.E-2	>1.E-2	**≤5.E-7** *	≥1.E-2
MID357-MID3774	896.139	>1.E-2	**≤5.E-7** *	>1.E-2	>1.E-2	>1.E-2	>1.E-2	>1.E-2

The results are sorted according to the distance between the two loci in the pair.

* Significant p-values of gametic association; **low p-values not considered significant after applying Bonferroni’s correction for multiple tests; ***No test was performed because one of the two loci was monomorphic in the sample. Note: the p-values were obtained for 100,172 Markov steps.

A significant association could be expected for MID356 and MID357, considering that these loci are just 5.2 Kb (0.0198 cM) apart at the X chromosome (see Table S1 in Pereira et al. [41]) and that significant *p-*values of LD were previously observed in Europeans and Africans [36]. Indeed, significant associations could be detected between the MID356 and MID357 alleles in all six samples from different Colombian regions (see Table 2). Notably, these markers are contiguous to two other markers, MID3703 and MID3774, separated by 97.7 Kb (0.26 cM). These two markers presented significant LD in the samples from the Central-East Andean region and showed low *p*-values in three other samples. The samples from the Central-East Andean region showed clear signs of association between all allele pairs inside a large block of 896,139 bp, including MID357, MID356, MID3703, and MID3774. This large linkage block was not observed in the African and European samples studied by Pereira et al. [36] or the other samples obtained from Colombia. Based on the results from the Hardy-Weinberg equilibrium test and AMOVA, no signs of population substructure were detected between the populations in the Central-East Andean region, suggesting more recent admixture events occurred in this region than in the other regions studied.

The close proximity of the loci MID3690, MID3719, and MID2089 (spanning approximately 174 Kb) suggests that these three markers might form a LD block [36], particularly in populations with a recent history of admixture. In the present study, significant values of gametic association were observed between the MID3690 and MID3719 loci in samples obtained from the Central-East Andean region, and between MID3719 and MID2089 in samples obtained from the Central-East Andean and Orinoquian regions and the North Pacific Coast. These results emphasise the need to treat MID3690, MID3719 and MID2089 as a haplotypic block.

For the remaining loci, no significant LD values were observed in most pairwise tests (data not shown). An exception was observed in the samples obtained from Nariño and Pastos (Table S4), where significant LD values were obtained between the 11 pairs analysed. These results are not correlated with the loci-pair distances and might reflect the small sizes of these two samples (26 and 15 unrelated males from Nariño and Pastos, respectively); therefore, the results for these two samples must be carefully interpreted.

The large LD block observed in the Central-East Andean region can be attributed to recent admixture.

### Analysis of Admixture

For the admixture analysis, we used previously published reference population data for Africans and Europeans [36]. Because no previous data were available for the studied markers in Native Americans, the data obtained from the Colombian Pastos group analysed in the present study were also used as a reference for Native Americans. A previous analysis of this population using autosomal SNP markers revealed low levels of European or African admixture [40].

**Figure 3:**
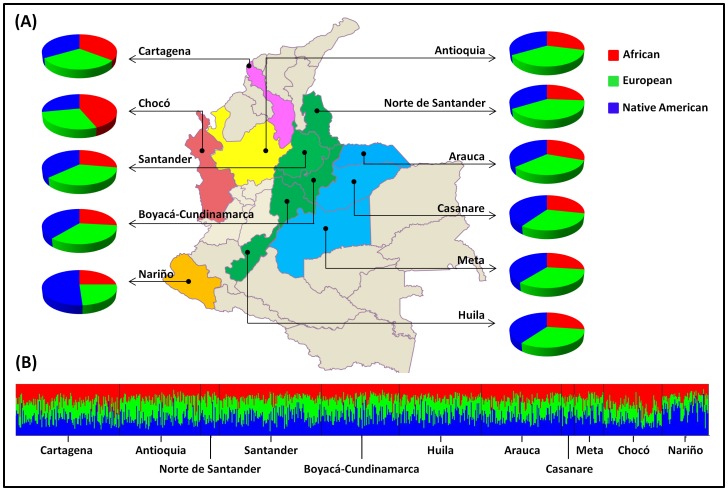
Schematic representation of the global population (A) and individual (B) admixture estimates (IAEs) in 11 Colombian admixed populations, using STRUCTURE v2.3.3 software (K = 3; parameter set details in Material and Methods), for African, European and Native American ancestry proportions.

The reference and study populations were simultaneously analysed to determine the ethnic contributions to all Colombian samples. The results obtained using the 32 X-indel polymorphisms showed similar African, European and Native American contributions to most regions (Figure 3A), except for the population from Chocó, on the North Pacific coast, which harbours the highest proportion of African admixture (44%). The highest Native American membership was observed in the Nariño region (51%). Slightly higher Native proportions were also observed in populations east of the Central-East Andean region (Santander, Cundinamarca-Boyacá and Huila) and the Orinoquian region (Arauca, Casanare and Meta) compared with the West (Antioquia). At an individual level (Figure 3B), we observed that although a significant variation among the individuals inside each population was observed, the three ancestral contributions were observed in all individuals.

### Comparison with Data from Autosomal Markers

Most South American populations were subjected to an intensive admixture, primarily comprising individuals from Europe and, to a lesser extent, Africa during the last 500 years.

Due to the inheritance properties of the X chromosome, mothers have a higher contribution to the offspring than the fathers. Therefore, in a population with the same number of males and females, two times more female than male ancestral contribution to the overall gene pool is expected after sex-biased admixture events [15].

To infer the gene flow patterns in the studied populations, we compared the interethnic admixture estimates obtained for the X-indels with those previously published for a group of 52 autosomal SNPs [40] for the three main ancestral contributors, after grouping the samples in six Colombian regions, and the results are indicated in Table 3.

**Table 3:** The interethnic admixture proportions using autosomal and X-chromosomal specific markers, considering three main ancestral contributors to the different regions of Colombia studied.

	Autosomal SNPs [40]			X chromosomal Indels (this study)		
Population	Africa	Europe	Native America	Africa	Europe	Native America
Caribbean Region	–	–	–	0.356	0.309	0.335
North Colombian Pacific Coast	0.538	0.230	0.232	0.437	0.282	0.281
Central-West Andean Region	0.211	0.443	0.346	0.290	0.375	0.335
Central-East Andean Region	0.196	0.415	0.389	0.273	0.345	0.382
Orinoquian Region	0.218	0.405	0.377	0.294	0.330	0.376
South-West Andean Region	0.188	0.301	0.511	0.250	0.239	0.511

In general, the results showed a lower contribution from European individuals to the X-chromosomal than to the autosomal gene pool, consistent with previous results on mtDNA and Y chromosome lineage markers, showing sex-biased mating between European men and Native and African women in many admixed populations throughout South America (e.g. [15], [23], [42]). Moreover, in most regions, we observed that the proportion of European contribution estimated from the autosomal and X-chromosomal gene pools is higher than that expected from averaging the proportions of mtDNA and Y chromosomal gene pools of European origin. These results were consistent with those of Bedoya et al. [15] and Rojas et al. [23], supporting a previous hypothesis of the posterior gene flow of individuals of European ancestry after the first admixture event involving European males and Native American females [15].

A more detailed regional analysis revealed that in the North Pacific coast, the apportionment of African ancestry was lower for X-indels than for autosomal SNPs, while for the European and Native American contributions, the inverse was observed. These findings and those from previous data for lineage markers [23], indicate that after the first sex-biased process of admixture, which occurred when the Europeans arrived to the North Pacific coast, a subsequent gender-biased gene flow occurred, in which males with high levels of African ancestry were admixed with females, having high Native American and European ancestries.

In the remaining regions, X-indels showed higher African and lower European contributions compared to autosomal SNPs; similar estimates were observed for the Native Americans. This pattern is consistent with a gene flow primarily involving males with higher European and lower African ancestry than the females, with no differences between Native American ancestry proportions in men and women.

## Conclusion

Comparing the results from different source markers can be important to disclose population events that act differentially in males and females. Therefore, 12 different Colombian samples were analysed using a set of 32 X-chromosomal indels, and the results were compared with previously reported data for 52 autosomal SNPs in Colombian populations [40]. The results obtained in the present study support those of previous studies obtained using autosomal markers, showing significant differences between different geographic regions in Colombia, particularly for those populations in the North Pacific and South-West Andean coastal regions. Nevertheless, Colombian populations are more similar in the X-chromosomal than the autosomal gene pool. Thus, the continental maternal ancestries of Colombians are more homogeneous than their paternal counterparts.

Notably, the African ancestry values estimated in the present study were higher than those previously reported [23]. Although this difference partially reflects different sampling strategies, we should also consider that the markers used in the present study are highly polymorphic in all continental population groups and, therefore, are not the most adequate to detect precise admixture proportions. Indeed, although these markers are capable of detecting the population substructure or relative differences in the ancestry of different population groups, the absolute ancestry values should be carefully interpreted.

The Chocó population (in the North Pacific coast) was least distant to the African reference population, and a slightly higher *F*_ST_ was obtained for the X-indels than for the 52 autosomal SNPs. The admixture analyses corroborate the results of the analysis of genetic distances, showing a higher African contribution for the autosomal than for the X-chromosomal markers. These results support a higher African ancestry of the males contributing to this population in contrast with a higher Native American ancestry of the females. A high African contribution was also detected in the population from the Caribbean region but, due to the lack of autosomal data, we could not confirm the same trend of a higher X-chromosomal than autosomal African ancestry. However, for the Caribbean region, Vergara et al. [22] reported a higher proportion of autosomal African ancestry than the average obtained using mtDNA and Y chromosome information. A biased mating between men with a higher African ancestry than women seems therefore to have occurred in both the Pacific and Caribbean coasts, which are the two regions with the highest African ancestry in Colombia. This high proportion of male-mediated African contribution is atypical of colonial settings and suggests that the admixture occurred during a period when Africans were no longer enslaved.

Data from lineage markers previously showed that Nariño, in the Southwest Andean region, has an almost complete Native American mtDNA gene pool and a predominance of Y-chromosomes from Europe [23]. In both autosomal and X-chromosomal studies, Nariño showed a high Native American ancestry. The similarity of the values calculated with autosomal and X-chromosomal markers suggests that an identical Native American ancestry of males and females contributed to the present-day population, after the first biased mating of European men and Native women, which nearly eradicated the European mitochondrial pool.

No significant differences in the interethnic admixture were observed among the remaining populations. Although a higher European ancestry was confirmed using autosomal markers, all populations presented identical European and Native American X-chromosomal contributions, which were slightly higher than the African contribution.

Clear signs of associations between the loci in a large block of 896,139 bp (MID357, MID356, MID3703, and MID3774) were observed in the sample from the Central-East Andean region, emphasising the importance of population admixture history in creating LD. Thus, in future studies, it would be interesting to increase the number of samples and populations analysed to assess the potential of this linkage block for dating admixture events in American populations.

## Supporting Information

Figure S1 **Shepard diagram for the two-dimensional MDS plot represented in** **Figure 2** **.** This Scatterplot shows the reproduced distances plotted on the vertical (y) axis versus the original values plotted on the horizontal (x) axis. The small deviation of the reproduced distances to the step-line indicates a good fit.
(PDF)

Table S1 **List of 32 X-indel genotypes from the samples included in the present study.**
(XLS)

Table S2 **Genetic distances (FST) between the Colombian populations (lower diagonal), Africa and Europe, and the corresponding non-differentiation p-values (upper diagonal).** Significant p-values are indicated in red, with a significance level of 0.0008 (after applying Bonferroni’s correction for multiple tests).
(DOCX)

Table S3 **Allele frequencies of 32 X-Indel markers in samples from a Native American group (Pastos) and from six Colombian regions: South-West Andean Region (Nariño); Central-West Andean Region (Antioquia); Central-East Andean Region (Boyacá-Cundinamarca, Huila and Santander); Orinoquian Region (Arauca, Meta and Casanare); North Colombian Pacific Coast (Chocó); Caribbean Region (Cartagena).** In the table it is represented the frequency of the shorter allele, called allele 1 (the frequency of allele 2 is 1 minus the frequency of allele 1, since each locus has only just two alleles).
(DOCX)

Table S4 **List of significant p-values of LD for polymorphic loci separated by more than 1 Kb.** The results are sorted according to the distances between the two loci in the pair.
(DOCX)
