# Supplementary material for: Evaluating the X Chromosome-Specific Diversity of Colombian Populations Using Insertion/Deletion Polymorphisms
Source: PLoS One. 2014 Jan 31;9(1):e87202. doi: 10.1371/journal.pone.0087202 (PMC3909073; doi:10.1371/journal.pone.0087202)

Supplementary Figure S1. Shepard diagram for the two-dimensional MDS plot represented in Figure 2. This Scatterplot shows the reproduced distances plotted on the vertical (y) axis versus the original values plotted on the horizontal (x) axis. The small deviation of the reproduced distances to the step-line indicate a good fit.

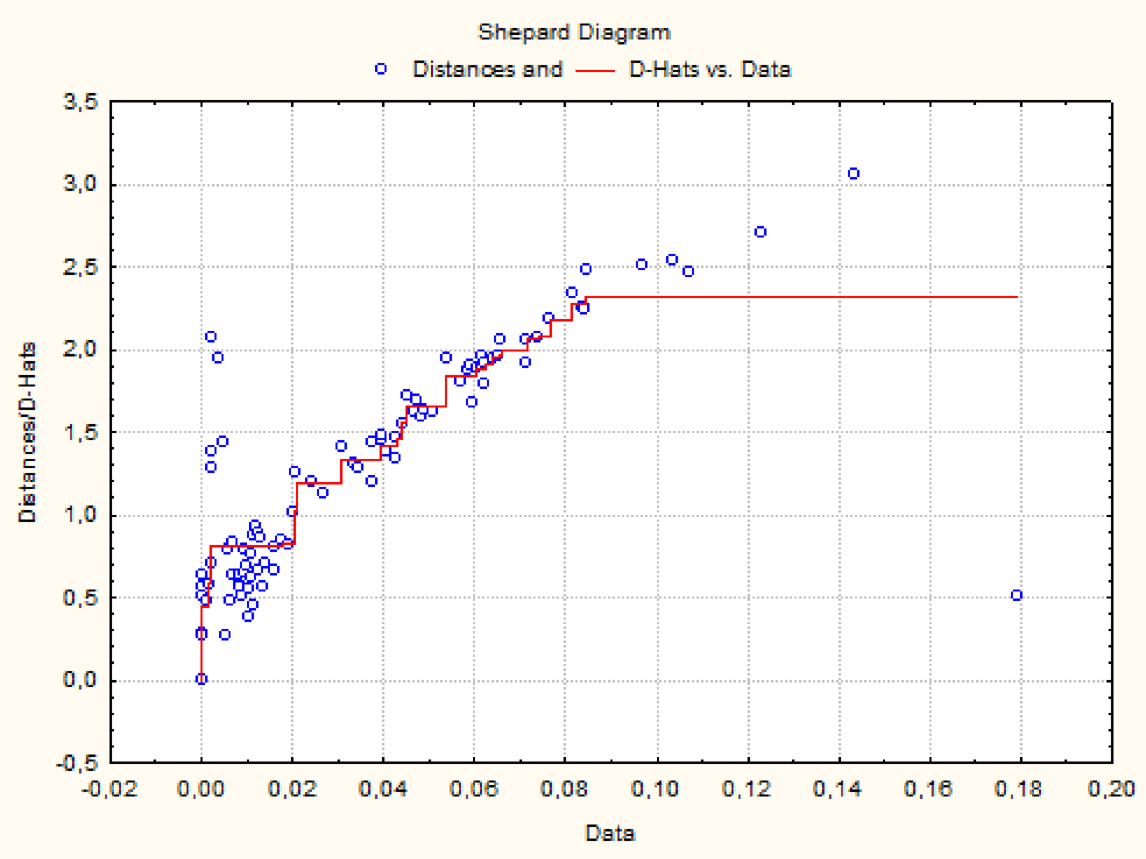

Supplement: Figure S1 — Shepard diagram for the two-dimensional MDS plot represented in Figure 2 . This Scatterplot shows the reproduced distances plotted on the vertical (y) axis versus the original values plotted on the horizontal (x) axis. The small deviation of the reproduced distances to the step-line indicates a good fit. (PDF) [file pone.0087202.s001.pdf]
