# Supplementary material for: Evaluating the X Chromosome-Specific Diversity of Colombian Populations Using Insertion/Deletion Polymorphisms
Source: PLoS One. 2014 Jan 31;9(1):e87202. doi: 10.1371/journal.pone.0087202 (PMC3909073; doi:10.1371/journal.pone.0087202)
Supplement: Table S2 — Genetic distances (FST) between the Colombian populations (lower diagonal), Africa and Europe, and the corresponding non-differentiation p-values (upper diagonal). Significant p-values are indicated in red, with a significance level of 0.0008 (after applying Bonferroni’s correction for multiple tests). (DOCX) [file pone.0087202.s003.docx]

**Supplementary Table S2.** Genetic distances (FST) between the Colombian populations (lower diagonal), Africa and Europe, and the corresponding non-differentiation p-values (upper diagonal). Significant p-values are indicated in red, with a significance level of 0.0008 (after applying Bonferroni’s correction for multiple tests).
