# Supplementary material for: Evaluating the X Chromosome-Specific Diversity of Colombian Populations Using Insertion/Deletion Polymorphisms
Source: PLoS One. 2014 Jan 31;9(1):e87202. doi: 10.1371/journal.pone.0087202 (PMC3909073; doi:10.1371/journal.pone.0087202)
Supplement: Table S3 — Allele frequencies of 32 X-Indel markers in samples from a Native American group (Pastos) and from six Colombian regions: South-West Andean Region (Nariño); Central-West Andean Region (Antioquia); Central-East Andean Region (Boyacá-Cundinamarca, Huila and Santander); Orinoquian Region (Arauca, Meta and Casanare); North Colombian Pacific Coast (Chocó); Caribbean Region (Cartagena). In the table it is represented the frequency of the shorter allele, called allele 1 (the frequency of allele 2 is 1 minus the frequency of allele 1, since each locus has only just two alleles). (DOCX) [file pone.0087202.s004.docx]

**Supplementary Table S3**

Allele frequencies of 32 X-Indel markers in samples from a Native American group (Pastos) and from six Colombian regions: South-West Andean Region (Nariño); Central-West Andean Region (Antioquia); Central-East Andean Region (Boyacá-Cundinamarca, Huila and Santander); Orinoquian Region (Arauca, Meta and Casanare); North Colombian Pacific Coast (Chocó); Caribbean Region (Cartagena). In the table it is represented the frequency of the shorter allele, called allele 1 (the frequency of allele 2 is 1 minus the frequency of allele 1, since each locus has only two alleles)

|  | **Locus** | **Native American group** | **South-West Andean Region** | **Central-West Andean Region** | **Central-East Andean Region** | **Orinoquian Region** | **Caribbean Region** | **North Colombian Pacific Coast** |
| --- | --- | --- | --- | --- | --- | --- | --- | --- |
| **MID3736** | rs56162621 | 0.1807 | 0.3095 | 0.5034 | 0.4289 | 0.3723 | 0.4691 | 0.6442 |
| **MID3730** | rs3215490 | 0.3012 | 0.3133 | 0.3724 | 0.2953 | 0.2554 | 0.2629 | 0.2500 |
| **MID1361** | rs2307557 | 0.0723 | 0.0952 | 0.1586 | 0.1250 | 0.1818 | 0.2577 | 0.2885 |
| **MID329** | rs25553 | 0.0120 | 0.0833 | 0.1448 | 0.1250 | 0.0693 | 0.1804 | 0.2115 |
| **MID3716** | rs63344461 | 0.4217 | 0.4167 | 0.6138 | 0.5690 | 0.5671 | 0.5361 | 0.5769 |
| **MID3692** | rs67929163 | 0.0361 | 0.0357 | 0.1724 | 0.1228 | 0.1255 | 0.2268 | 0.3558 |
| **MID2637** | rs3053615 | 0.9759 | 0.9643 | 0.9379 | 0.9375 | 0.9307 | 0.8969 | 0.8846 |
| **MID3740** |  | 0.1566 | 0.2976 | 0.3034 | 0.3341 | 0.2424 | 0.3711 | 0.3173 |
| **MID198** | rs16637 | 0.7229 | 0.6667 | 0.6690 | 0.6422 | 0.6797 | 0.6134 | 0.5577 |
| **MID3703** | rs59400186 | 0.2530 | 0.3133 | 0.2276 | 0.2392 | 0.1558 | 0.2938 | 0.2596 |
| **MID3690** | rs60283667 | 0.5542 | 0.5000 | 0.4552 | 0.4332 | 0.4372 | 0.3711 | 0.2524 |
| **MID3722** |  | 0.1566 | 0.1190 | 0.2414 | 0.2349 | 0.2684 | 0.2938 | 0.2981 |
| **MID3732** | rs11277082 | 0.1085 | 0.1190 | 0.2552 | 0.1897 | 0.2468 | 0.2577 | 0.2981 |
| **MID3712** | rs55877732 | 0.5422 | 0.4643 | 0.2828 | 0.3147 | 0.2554 | 0.4072 | 0.4135 |
| **MID1736** | rs2307932 | 0.1446 | 0.1190 | 0.2483 | 0.2909 | 0.2511 | 0.2268 | 0.1346 |
| **MID3719** | rs3078486 | 0.1446 | 0.2143 | 0.2138 | 0.2294 | 0.2096 | 0.3037 | 0.3462 |
| **MID2089** | rs3028280 | 0.2410 | 0.3929 | 0.2828 | 0.3268 | 0.2987 | 0.4227 | 0.4904 |
| **MID3774** | rs5901519 | 0.7349 | 0.6310 | 0.4414 | 0.5280 | 0.5281 | 0.5412 | 0.5673 |
| **MID3760** | rs66676381 | 0.7470 | 0.7108 | 0.8138 | 0.7446 | 0.7229 | 0.8351 | 0.7981 |
| **MID3701** | rs4030406 | 0.4578 | 0.5833 | 0.4621 | 0.5323 | 0.5801 | 0.5928 | 0.5962 |
| **MID2612** | rs3048996 | 0.1084 | 0.1905 | 0.4069 | 0.3664 | 0.3680 | 0.3608 | 0.3654 |
| **MID1839** | rs2308035 | 0.3012 | 0.3690 | 0.4759 | 0.4902 | 0.5108 | 0.4794 | 0.4327 |
| **MID3754** | rs57608175 | 0.8434 | 0.6548 | 0.3931 | 0.4461 | 0.4805 | 0.5103 | 0.5385 |
| **MID111** | rs16397 | 0.7349 | 0.7381 | 0.6483 | 0.7002 | 0.6840 | 0.5773 | 0.4423 |
| **MID2652** | rs3080039 | 0.5663 | 0.5952 | 0.6552 | 0.6228 | 0.6494 | 0.5053 | 0.5481 |
| **MID1511** | rs2307707 | 0.5904 | 0.5714 | 0.7310 | 0.6573 | 0.6104 | 0.4691 | 0.2692 |
| **MID2692** | rs3047852 | 0.3012 | 0.3494 | 0.5034 | 0.4655 | 0.4805 | 0.4560 | 0.3077 |
| **MID357** | rs25581 | 0.6867 | 0.5833 | 0.3586 | 0.5043 | 0.4329 | 0.4278 | 0.2788 |
| **MID356** | rs25580 | 0.7108 | 0.5952 | 0.4000 | 0.5325 | 0.4502 | 0.4323 | 0.2692 |
| **MID243** | rs16680 | 0.9518 | 0.9167 | 0.8828 | 0.9073 | 0.8312 | 0.8814 | 0.8558 |
| **MID3727** | rs3050111 | 0.2651 | 0.2262 | 0.2207 | 0.2112 | 0.3074 | 0.3402 | 0.3558 |
| **MID3753** | rs72417152 | 0.0120 | 0.0357 | 0.1034 | 0.1277 | 0.1179 | 0.1392 | 0.0962 |
