# Supplementary material for: Evaluating the X Chromosome-Specific Diversity of Colombian Populations Using Insertion/Deletion Polymorphisms
Source: PLoS One. 2014 Jan 31;9(1):e87202. doi: 10.1371/journal.pone.0087202 (PMC3909073; doi:10.1371/journal.pone.0087202)
Supplement: Table S4 — List of significant p-values of LD for polymorphic loci separated by more than 1 Kb. The results are sorted according to the distances between the two loci in the pair. (DOCX) [file pone.0087202.s005.docx]

**Supplementary Table S4.** List of significant *p*-values of LD for polymorphic loci separated by more than 1 Kb. The results are sorted according to the distances between the two loci in the pair.
